# Inhibitory activity of FOXP3+ regulatory T cells reveals high specificity for displaying immune tolerance in remission state rheumatoid arthritis

**DOI:** 10.1038/s41598-020-76168-1

**Published:** 2020-11-13

**Authors:** Korawit Kanjana, Parawee Chevaisrakul, Ponpan Matangkasombut, Karan Paisooksantivatana, Putthapoom Lumjiaktase

**Affiliations:** 1 Department of Pathology, Faculty of Medicine Ramathibodi Hospital, Mahidol University, Rama 6 Road, 270 Thung Phaya Thai, Ratchathewi, Bangkok, 10400 Thailand; 2 Division of Allergy, Immunology and Rheumatology, Department of Medicine, Faculty of Medicine Ramathibodi Hospital, Mahidol University, Bangkok, Thailand; 3 Department of Microbiology, Faculty of Science, Mahidol University, Bangkok, Thailand; 4 Division of Rheumatology, Allergy and Immunology, Center for Immunology and Inflammatory Diseases, Massachusetts General Hospital, Harvard Medical School, Boston, MA USA

**Keywords:** Immunology, Biomarkers, Diseases, Rheumatology

## Abstract

Immune regulation status may indicate immunological remission in rheumatoid arthritis (RA). This cross-sectional study aimed to determine the Regulatory T cell (Treg) properties, together with 14 plasma cytokines levels between active RA and clinical remission patients. Peripheral blood (PB) Foxp3+ Treg was collected from RA patients for determination of Treg inhibitory activity using a co-culture system. Other PB T cell types and plasma cytokines were determined by flow-cytometry. The Treg results were analyzed according to the disease activity score-28 (DAS28). Then sensitivity and specificity were calculated for the indication of the remission status. The number and inhibitory activity of Treg are higher in the clinical remission as compared to the active RA (*p* value < 0.0001). Also, Treg: CD4+CD25+CD127+ cell ratio demonstrates the similar result (*p* value < 0.05). Treg inhibitory activity is inversely correlated with the DAS28. Specificity and positive likelihood ratio of inhibitory activity for indicating remission status are 92.31% (95% CI 63.97–99.81) and 11.14 (95% CI 1.67–74.14), respectively. Treg inhibitory activity is a promising prognostic marker and probably represents the immunological remission status in RA.

## Introduction

Rheumatoid arthritis (RA) is a systemic autoimmune disease, manifesting mainly as joint inflammation. RA severity can be evaluated in several ways, one of which being a disease activity score-28 (DAS28 score), number of damaged joints and erythrocyte sedimentation rate (ESR) or C-Reactive Protein (CRP), with an RA patient having DAS28 score < 2.6 considered in a remission state and > 2.6 in an active state^1^. RA patients in remission can be categorized into those with clinical, with imaging/serological and with immunological remission states^2^. Immunological remission may reflect a condition of restored immunological status, e.g. normal cytokines level and response of inflammatory cells. In seropositive RA, rheumatoid factor (RF) could turn to be negative after controlled inflammation, although anti-citrullinated protein antibodies (ACPA) is always positive even after clinical remission. However, RA patients with immunological remission according to the above-mentioned definition are hardly ever found and the ideal treatment for this state remains challenging^2^. A long-term monitoring of ACPA/RF level in early arthritis patients showed alterations to this ratio are not associated with outcome parameters^3^.

Foxp3+ regulatory T cell (Treg) is a major immune cell suppressor. Its number is decreased among patients in RA active state^4,5^, but Treg numbers do not indicate the balance in immune status because of cytokine-T cell plasticity^6^. Although functional studies of Treg should provide insights of immune status in RA, most studies contain only a small population of subjects to provide data of statistical significance regarding Treg suppression or inhibition properties^7–9^. In addition, the use of CD4+ and CD25+ markers for Treg isolation can include other types of activated T cells as CD25 is expressed in these cell types^10^. Isolation of Treg cells requires the use of unique marker, fulfilled by CD4+CD25high+CD127low− population, which strongly correlates with Foxp3+ Treg phenotype^11,12^.

To display the whole immunological tolerance status, this study measured Foxp3+ Treg inhibitory activity, frequencies of Foxp3+ Treg and effector T cell, and cytokine levels from peripheral blood of clinical remission RA and active state RA patients.

## Methods

### Participants and study design

The study was carried out in accordance with international guidelines and declaration of Helsinki, which was approved by the Ethics Committees, Faculty of Medicine Ramathibodi Hospital, Mahidol University. Prior written informed consent were obtained from all individual participants and for participants below age of 18 years, parental informed consent was obtained.

Rheumatoid arthritis patients (*n* = 27) according to ACR/EULAR 2010 or ACR 1987 classification^13^ and attending at the Rheumatology Clinic, Faculty of Medicine Ramathibodi Hospital, Mahidol University, Bangkok, Thailand were enrolled to the study. Inclusion criteria are RA patients of 15–65 years of age and follow-up at the Rheumatology Clinic for at least 2 years; and exclusion criteria were patients with a history of infection during at least two weeks prior to the study or with underlying diseases such as cancer and immune deficiency. Healthy volunteers (n = 7) were those with white blood cell count within normal limit and no history of any underlying diseases and use of immunosuppressant drugs.

Patient classifications: (1) The RA patients were grouped into two groups, following; clinical remission RA and active RA, based on their DAS28 score and/or clinical symptoms. DAS28 score is calculated based on the number of tender joint and swollen joint inflammation and ESR. The clinical remission RA (n = 14) is defined when DAS28 score < 2.6 or without detected clinical synovitis (absence of pain symptom and swollen or tender joint) sustained for at least six months. In this remission RA group, three of them had low disease activity (DAS28 score; 2.63–2.71) but without detected clinical synovitis. Active RA (n = 13) is defined by DAS28 score > 2.6 or presence of clinical symptoms and ESR > 50 mm/hr or CRP > 15 mg/L. (2) The RA patients were classified based on their DAS28 score, patients who had DAS28 score < 2.6 was defined as remission (n = 11), ≥ 2.6–3.2 was defined as low DAS28 (n = 8), ≥ 3.2–5.0 was defined as moderate DAS28 (n = 6), and ≥ 5.1 was defined as high DAS28 (n = 2). This classifications were applied for analysis when the initial data between clinical remission RA and active RA was significantly difference. (3) All patients were also categorized based on their weekly different doses of Methotrexate (MTX), including: patients who received < 10 mg/week (n = 17), 11–17.5 mg/week group (n = 6) and ≥ 20 mg/week (n = 4). This classification was used for analyzing the Treg inhibitory activity in different doses of MTX used. Other conventional DMARDs would not be classified because the patient received different DMARDs regimen. (4) To determine the alteration of Treg inhibition in the same person, The Treg determination were done in three patients (*n* = 3) who were followed-up at active state and entered into remission RA. The inclusion criteria and defining state of these patients are the same as the definition of the active RA and remission RA as described above.

### Materials and reagents

In our previous study (12), the fluorescence activated cells were sorted by flow cytometer (FACS Aria III, Becton Dickinson, USA). The determination of CFSE proliferation was measured by Flow cytometer (FC500, Beckman Coulter, USA). CD8 T cell was depleted by X-Zell Biotech kit (Thailand). The materials and reagents for used with this work included recombinant human Intereukin-2 from ImmunoTools (Germany), Ethylenediaminetetraacetic acid (EDTA) and heparinized BD vacutainer CPT tube from Becton Dickinson (USA), 96-well microtiter plates, penicillin–streptomycin solution, 500 mL phosphate-buffered saline (PBS) pH 7.4, hydroxyethyl piperazineethanesulfonic acid (HEPES), sodium pyruvate anhydrous, 2-mercaptoethanol and Trypan Blue solution 0.4% were from Sigma-Aldrich (USA), glutamine-supplemented RPMI-1640 medium, carboxyfluorescein succinimidyl ester (CFSE) proliferation kit, fetal bovine serum (FBS) and anti-human CD3/CD28 bead (cat 111.31D) from Life Technologies (USA), PE-Cy7 Mouse Anti-Human CD4 (cat.557852), APC Mouse Anti-Human CD8 (cat.340584), PE-Cy5 Mouse Anti-Human CD25 (cat.555750) and PE Mouse Anti-Human CD127 (cat.557938) from Becton Dickinson (USA), and eFluor660 Anti-Foxp3 (236A/E7), fixing/permeabilizing solution from Affymetrix eBioscience (USA). The serology test which contribute to the diagnosis of RA is that for the quantitative determination of RF in serum (range 10–130 IU/mL) on Roche Cobas C501 (Switzerland) and the semi-quantitative determination of ACPA in serum (range 0.5–200 U/mL) on ARCHITECT Abbott Laboratory (USA).

### Cell sorting and Foxp3 Treg, T cell subset identification

In brief, the plasma from fresh EDTA-blood of each participant was prepared by centrifuge at 1500xg, 4 °C for 5 min and stored at − 80 °C until assayed for cytokines. Peripheral blood mononuclear cells (PBMCs) were isolated using the BD Vacutainer CPT tube for PBMC isolation (centrifuged at 1700×*g*, 20 °C for 20 min). After washed twice with PBS that PBMC (10–15 × 10^6^) were incubated with anti-CD8 antibodies, carried by the magnetic column kit for CD8 depletion (X-Zell Biotech, (Thailand)), for 5 min, then washed with PBS, centrifuged at 450×*g* for 10 min at 4ºC, and incubated with nano-magnetic bead reagent for 10 min. After that, the cells were suspended with 1 mL of the commercial kit buffer, transferred to flow-through CD8+ cell depletion employing the commercial kit magnetic column (X-Zell Biotech, (Thailand)). The CD4+ enrichment was obtained and centrifuged at 500 g for 10 min at 4ºC. Following the CD4+-enriched cells were treated with mouse anti-human fluorescent dye-conjugated antibodies [CD4PEcy7, CD25PEcy5 and CD127PE (Becton Dickinson, USA)] for cell sorting and isolation of Treg (CD4+CD25high+CD127low−) and conventional T cell or Tconv (CD4+CD25−CD127+) with FACSAria III. Both T cell populations were used for co-culture inhibition assay.

**Figure 1:**
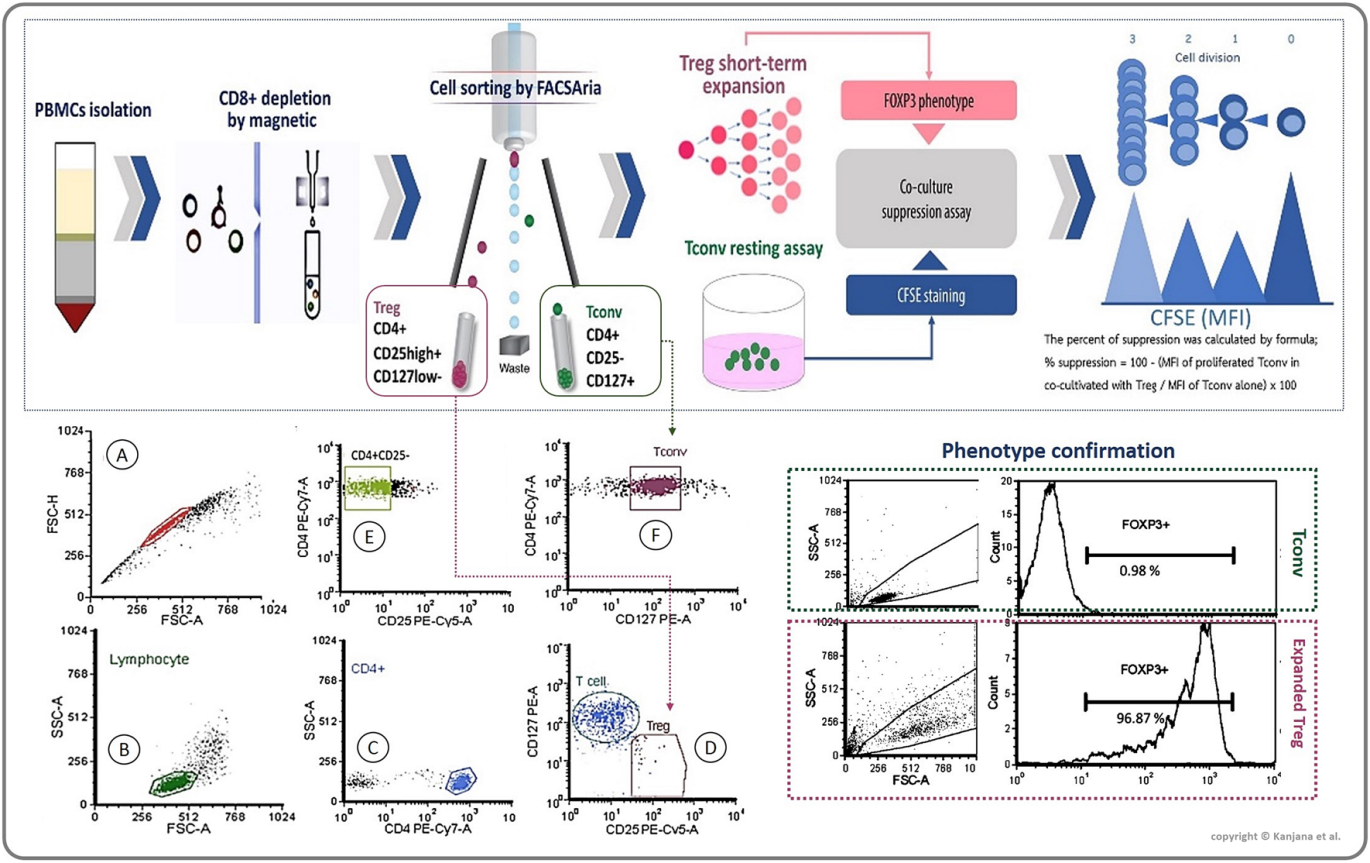
Method framework and flowcytometry gating strategies. Peripheral blood mononuclear cells (PBMCs) were isolated by their intensity-centrifugation, followed by CD8+ cell depletion employing a magnetic column method. CD4+-enriched cells were stained with mouse anti-human fluorescent dye-conjugated antibodies [CD4PEcy7, CD25PEcy5 and CD127PE for isolation of Treg (CD4+CD25high+CD127low−) and conventional T cell or Tconv (CD4+CD25−CD127+) with FACSAria III. The gating strategies started from debris discrimination (**A**), followed by lymphocyte (**B**) and CD4+ gating (**C**). Treg population (**D**) and Tconv gating were shown (**E**, followed by **F**). Short-term Treg expansion assay was performed for gaining Treg cell number. Beside, Tconv cells were rested by resting assay parallel with the Treg expansion assay for four days at 37 °C under 5% CO_2_ atmosphere. Followed by phenotype checking on Treg and Tconv by intracellular Foxp3+ expression using flow-cytometry method. Afterward, Carboxyfluorescein succinimidyl ester (CFSE) was stained in Tconv population for following-up T cell proliferation. Followed by Treg co-culture assay for three days at 37 °C under 5% CO_2_ atmosphere, under stimulation of anti-CD3/28 beads and supplemented by 500 IU of Interleukin 2. CFSE stained Tconv proliferation was determined by flowcytometry. The formula for percent Treg inhibition was provided in the figure. This figure was created officially by first author.

In order to determine by flow-cytometry, the numbers of Foxp3+ Treg cells and T cell subset originally present in whole blood, PBMCs without CD8+ depletion were stained for presence of intracellular protein Foxp3 with rat fluorescent dye-conjugated anti-human Foxp3eflu660. CD4+CD25+Foxp3+ population is defined as Foxp3+ Treg (intra-maker Treg) while T cell sub-populations were extracellular stained as CD4+CD25+CD127+ (activate T cells) and CD4+CD25high+CD127low− (extra-maker Treg) populations. In addition, both ratio of Foxp3 Treg:CD4+CD25+CD127+ population (Foxp3+ Treg ratio) and CD4+CD25high+CD127low−:CD4+CD25+CD127+(CD4+CD25high+CD127low− ratio) were performed. Overall protocol steps and gating strategies were demonstrated in Fig. 1. Full descriptions of the protocol is given in our previous study (12).

### Autologous co-culture inhibition assay

Short-term of Treg (CD4+CD25high+CD127low−) cell expansion assay (10^4^ cells/well) were incubated with culture medium supplement with 500 IU/mL recombinant human interleukin 2 (rIL-2) and anti-human CD3/CD28 bead at a ratio 3:1 bead: cell for four days at 37 °C under 5% CO_2_ atmosphere. Treg phenotype was confirmed by staining of intracellular Foxp3 with rat fluorescent dye-conjugated anti-human Foxp3eflu660. All preparations contained > 90% Foxp3 positive cells (Phenotype confirmation, Fig. 1). Tconv was maintained (rested) in culture medium supplemented with 500 IU/mL rIL-2 for four days. Rested Tconv cells were stained with CFSE prior to co-culturing with autologous expanded Treg cells at Tconv:Treg cell ratio of 1:10, 1:5, and 1:0 (Tconv alone). A 500 IU/mL aliquot of rIL-2 together with anti-CD3/CD28 beads (1:2 bead:cell) were added to the co-culture solution and incubated at 37 °C for three days under 5% CO_2_ atmosphere. Then, CFSE-stained Tconv proliferation was determined by flow-cytometry. Percent Treg inhibitory activity of Treg was calculated as follows: percent inhibition = 100 − [(percent Tconv proliferation in co-culture)/(percent Tconv proliferation in absence of Treg)] × 100. The details of protocol validation in healthy individuals and RA patients is given in our previous study^12^.

### Cytokines determination

The cytokines detection was done by using the BioLegend LEGENDplex protocol (Cat. No. 740723, Human Th1 Cytokine Panel (13-plex) with Filter plate; 13 human cytokines including IL-2, 4, 5, 6, 9, 10, 13, 17A,17F, 21, 22, IFN-Ƴ and TNF-α) recommendation. The range limit of detection is 0–10,000 pg/ml. Briefly, the stored plasma EDTA were thawed completely under room temperature. All plasma was diluted by twofold dilution with assay buffer prior to assay. This experiments are done in triplicate. The plasma were incubated with capture beads and detection antibody in a rotator for 2 h at room temperature. After that, SA-PE reagent was added into the sample tubes and incubated for 30 min, washed twice and kept in dark until determined by flow cytometer.

### Statistical analysis

Descriptive results are presented as mean (± standard deviation, SD), median and percentage. Corresponding inferential comparisons of Treg inhibitory activity, frequencies of Treg and T cell subset, and plasma cytokine level were calculated using *t*-test (for parametric data) and Mann–Whitney *U* test or Kruskal Wallis test (for non-parametric data). Skewness and Kurtosis were used for data distribution analysis. Cross-tabulation analysis was performed for the calculation of sensitivity, specificity, Positive Predictive Value (PPV), Negative Predictive Value (NPV), Positive Likelihood Ratio (LR+) and Negative Likelihood Ratio (LR-). Statistical analysis was carried out using IBM SPSS version 16.00 for Window OS (SPSS Inc., USA). A *p* value < 0.05 is considered statistically significant.

## Results

### Patient’s characteristics

The average age of the RA patients is not different between those in clinical remission and active RA (Table 1). All 14 patients in the clinical remission group were female while only one male was present in the active disease group. In the former group, the range of DAS28 scores were lower than the latter group. The majority of RA patients in remission was symptom-free for > 6 months prior to the recruitment. Three patients were symptom-free for > 2 years. VAS score were 0–30 in the RA remission group and 0–60 in the active RA group. Serology test (RF or ACPA level) was positive for 64% of remission and 85% of active RA groups. The majority of both groups were treated with three-DMARDs and the remaining with two. In the remission group, only one patient was treated with methotrexate alone and no NSAID was prescribed. No biological DMARDs used in all patients. The patients were categorized into four group depending on their DAS28 score as described above. The remission (n = 11), low (n = 8), moderate (n = 6), and high DAS28 (n = 2) groups have their DAS28 score (median ± IQR) followed; 2.24 ± 1.02, 2.75 ± 0.30, 3.90 ± 0.97, and 4.84 ± 0.41, respectively. There was no statistical difference in DAS28 score between the negative *vs* positive serology patients (2.3 ± 0.7 *vs* 3.2 ± 1.4, *p* value = 0.111), especially when separated the patients based on DAS28 score as described above. The DAS28 scores between negative and positive serology in the remission and the low-DAS28 score group were 2.0 ± 0.7 versus 1.9 ± 0.6 (*p* value = 0.541) and 2.6 ± 0.0 *vs* 2.8 ± 0.2 (*p* value = 0.899), respectively. However, all patients in the moderate and high-DAS28 score groups had positive serology results (Supplementary Table 1). In addition, the correlation of DAS28 score and serology was performed (r = 0.311, *p* value = 0.115). The ESR values (median ± IQR) between the negative and positive serology patients were 24.0 ± 13.3 and 21.7 ± 14.18 mm/h, *p* value = 0.768. For the three patients who were followed-up at active state and after entered into remission RA, DAS28 score at active state and remission state are 2.99 ± 0.18 and 1.95 ± 0.62, respectively. The age-range is 45–67 years and one of them has negative serology.

**Table 1:** Rheumatoid arthritis (RA) patients’ characteristics.

Parameters	Healthy Median ± IQR	Remission RA Median ± IQR	Active RA Median ± IQR	*p* value^#^
*n* (%)	7	14 (51.9%)	13 (48.1%)	***–***
Age	30 ± 3	57 ± 22	56 ± 19	0.776
Female (%)	71	100	92	0.334
WBC (cell/mm^3^)	6.94 ± 2.11	5.97 ± 2.69	6.59 ± 3.30	0.913
Lymphocyte (%)	30.29 ± 7.00	28.50 ± 9.25	26.00 ± 9.00	0.076
ESR (mm/h)	***–***	28.00 ± 23.75	54.00 ± 65.00	0.013*
ACPA/RF positivity (%)	***–***	64.29	84.62	0.007**
VAS (range)	–	0^a^, 10–30^b^	0–60	0.000***
DAS28 score	–	2.33 ± 1.94	3.78 ± 1.73	0.000***
Duration of remission (%)	–	14 (< 6 months); 64 (6–24 months); 22 (> 24 months)	–	–
**Treatment (%)**				
Mono-therapy	–	7	–	–
Two DMARDs	–	36	40	–
Three DMARDs	–	57	60	–

*ACPA* anti-citrullinated protein antibodies, *DAS28* disease activity score-28, *DMARD* disease-modifying antirheumatic drug, *ESR* erythrocyte sedimentation rate, *RF* rheumatoid factor, *VAS* visual analog scale, *WBC* white blood cell count.

Significance at *p value < 0.05, **p value < 0.01, ***p value < 0.0001.

^#^Comparison of remission and active RA.

^a^Remission state patients (n = 11).

^b^Low disease activeity with clinical remission (n = 3).

### Percent Foxp3 Treg and T cell sub-populations in the whole blood

Intracellular Foxp3 staining was performed for Treg identification and quantification by flow-cytometry. The average percentage of Foxp3 Treg from the total CD4+ T cells in clinical remission group was higher than those of the active RA group, *p* value < 0.001 (Table 2). In addition, percentage (Median) of Foxp3 Treg cells were 3.07 ± 1.57, 2.35 ± 1.85, 2.09 ± 1.09, and 2.64 ± 1.23 in patients with DAS28 score < 2.6 (remission), ≥ 2.6–3.2 (low DAS28 score), ≥ 3.2–5.0 (moderate DAS28 score; active RA), and ≥ 5.1 (high DAS28 score; active RA), respectively. However, a significant difference (*p* value < 0.05) in percentage of Foxp3 Treg cells was found only between remission and moderate DAS28 score groups (Supplementary Figure S1). Among the other T cell sub-populations, the only significant finding was the lower average percent of CD4+CD25+CD127+ cells in the remission group as compared to RA active group. Additionally, both Foxp3+ Treg ratio and CD4+CD25high+CD127low− ratio were higher in the remission as compared to the active RA group, *p* value < 0.05 (Table 2). We found that there are higher Treg and ratios in healthy controls, but lower active T cells, indicating that healthy controls have more regulatory function when compared to active RA. Moreover, the percentage of Foxp3+ Treg and CD4+CD25high+CD127low− were not significantly different when compared with remission RA. These findings consisted that the both parameters in remission RA were not different when compared to healthy controls. Besides, even the CD4+CD25high+CD127low− population in active RA was lower than the healthy controls, but it was not significantly different (Table 2). In addition, the positive correlation (*r* = 0.878) of Foxp3+ Treg ratio and CD4+CD25high+CD127low− ratio in all patients was shown in Supplementary Figure S2.

**Table 2:** Immunological biomarkers of rheumatoid arthritis (RA) patients.

Parameters	Healthy controls (HC) Median ± IQR (n = 7)	Remission RA group (RR) Median ± IQR (n = 14)	Active RA group (AR) Median ± IQR (n = 13)	*p* value		
				HC vs RR	HC vs AR	AR vs RR
**T cell sub-population (% of CD4+ T cells)**						
Foxp3+ Treg	3.95 ± 0.55	3.07 ± 1.47	2.01 ± 0.82	0.138	0.003**	0.001**
CD4+CD25high+CD127low−	4.37 ± 1.34	3.69 ± 0.95	2.92 ± 2.24	0.195	0.183	0.132
CD4+CD25+	2.28 ± 2.51	2.71 ± 8.88	12.44 ± 21.10	0.004**	0.009**	0.356
CD4+CD25+CD127+	1.14 ± 2.04	7.70 ± 7.73	13.48 ± 11.31	0.008**	0.018*	0.043*
Foxp3+ Treg ratio^a^	3.83 ± 4.95	0.46 ± 0.83	0.14 ± 0.52	0.010*	0.009**	0.033*
CD4+CD25high+CD127low− ratio^b^	3.54 ± 4.88	0.66 ± 0.95	0.34 ± 0.38	0.014*	0.009**	0.044*
**Inhibitory activity#**						
% Inhibition	64.18 ± 2.96	57.24 ± 18.11	23.67 ± 10.94	0.448	< 0.0001***	< 0.0001***
**Suppressive cytokine**						
TGF-β (ng/mL)	27.09 ± 70.23	237.6 ± 183.9	304.3 ± 157.9	0.002**	0.001**	0.087
IL-10 (pg/mL)	38.70 ± 15.71	47.7 ± 15.7	68.3 ± 27.7	0.853	0.013*	0.049*
**Pro-inflammatory cytokine (pg/mL)**						
IL-2	4.12 ± 1.93	2.9 ± 1.7	6.9 ± 7.4	0.230	0.339	0.744
IL-4	34.66 ± 36.87	36.3 ± 27.4	87.6 ± 105.4	0.308	0.055	0.458
IL-5	< 1.9 (all samples)	< 1.9 (all patients)	< 1.9 (all patients)	–	–	–
IL-6	1.87 ± 0.82	4.7 ± 3.5	12.6 ± 10.8	0.109	0.028*	0.006**
IL-9	3.92 ± 2.76	3.1 ± 1.2	6.5 ± 7.7	0.378	0.093	0.694
IL-13	1.87 ± 0.88	2.6 ± 2.5	5.3 ± 6.4	0.473	0.121	0.316
IL-17A	1.94 ± 1.40	3.0 ± 1.5	11.7 ± 12.0	0.454	0.002**	0.009**
IL-17F	1.98 ± 5.36	4.5 ± 2.4	5.0 ± 2.3	0.541	0.382	0.189
IL-21	12.51 ± 12.21	11.5 ± 7.1	21.1 ± 30.1	0.405	0.190	0.965
IL-22	40.06 ± 29.97	40.7 ± 16.7	44.0 ± 21.0	0.781	0.861	0.827
INF-γ	12.04 ± 10.80	11.5 ± 5.8	19.2 ± 15.1	0.926	0.055	0.239
TNF-α	12.59 ± 15.48	24.1 ± 12.9	55.1 ± 68.8	0.459	0.045*	0.033*

^a^Foxp3+ Treg:CD4+CD25+CD127+.

^b^CD4+CD25high+CD127low−:CD4+CD25+CD127+.

Significance at *p value < 0.05, **p value < 0.01, ***p value < 0.0001 (Independent *t-*test and Mann–Whitney *U* test).

^#^Reported by mean ± SD, *IL* interleukin, *INF-γ* interferon gamma, *TGF-β* transforming growth factor beta.

### Inhibitory activity of Treg cells

**Figure 2:**
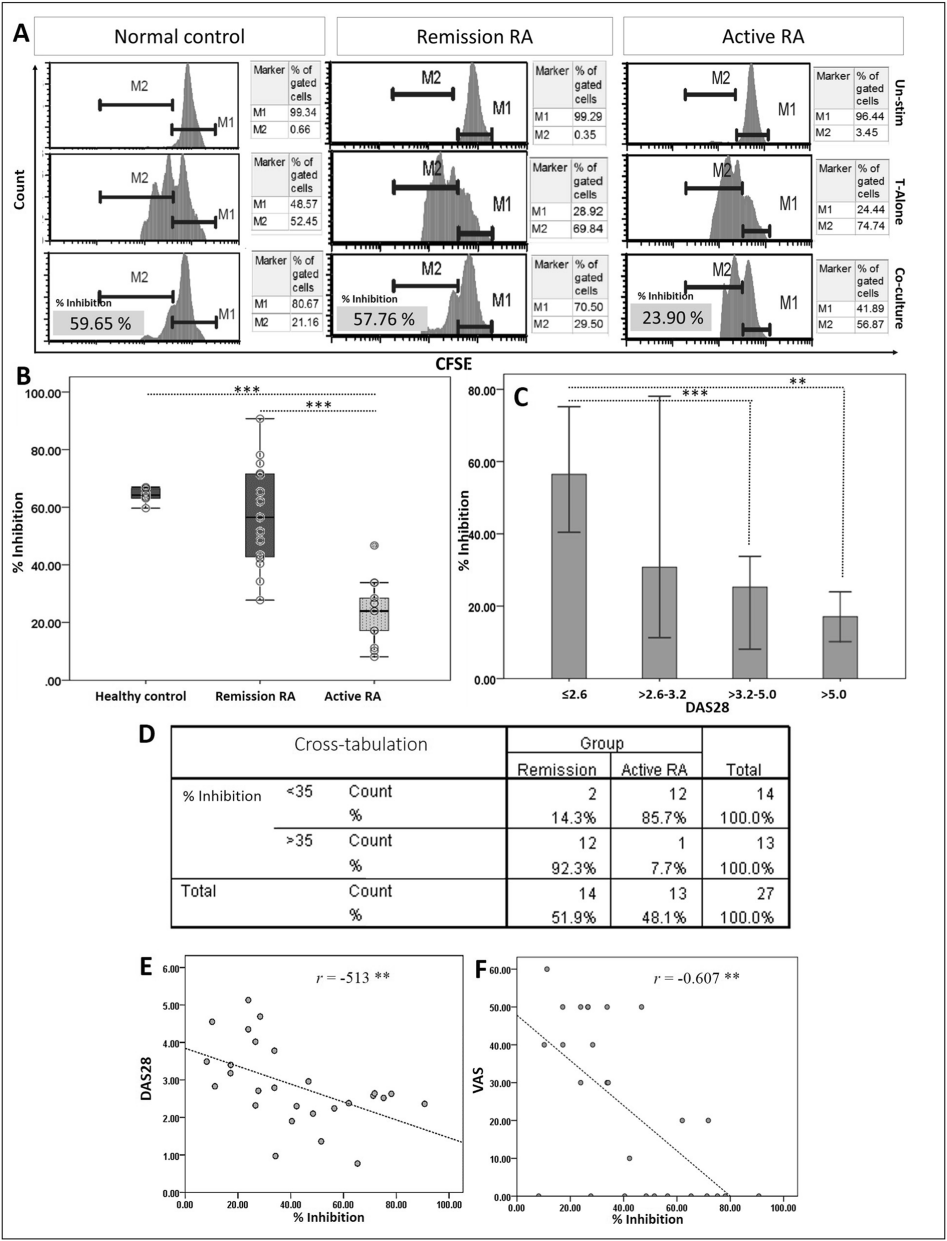
(**A**) Histogram analysis in such healthy control and different states of RA (n = 1 for each state). M (marker) 1 is % CFSE of undivided Tconv (an original peak from day 0 of proliferation); M2 is % CFSE of proliferated Tconv. (**B**) Treg inhibitory activity of in different states of RA (*n* = 14; remission, *n* = 13; active state) and (**C**) in patients categorized by active states according to disease activity score-28 (DAS28) score. Inhibitory activity of Foxp3+ Treg was measured after 3 days co-culture with Tconv cells by flow-cytometry. DAS28 score < 2.6, remission (*n* = 11); ≥ 2.6–3.2, low disease activity (*n* = 8); ≥ 3.2–5.0, moderate disease severity (*n* = 6); ≥ 5.1, high disease severity (*n* = 2). **p value < 0.01, *** p value < 0.0001 (independent *t*-test and Kruskal Wallis test). (**D**) Cross-tabulation analysis for calculating sensitivity, specificity and others indicating RA status statistic. (**E** and **F**) The correlation between Treg inhibitory activity and DAS28 score and VAS score (tested by Pearson correlation).

Foxp3 Treg following a short-term expansion period and concurrently rested Tconv cells were co-cultivated for three days. Histograms of inhibitory activity analysis in healthy control, remission RA and active RA patients of Tconv:Treg ratio at 1:10 were shown in Fig. 2A, which each state composes of unstimulating, Tconv stimulating alone and co-culture of Tconv with Treg stimulating. Moreover, the others histogram of inhibitory activity in varying Tconv:Treg dilutions were also demonstrated (Supplementary Figure S3A). Percent inhibition by Foxp3 Treg cells was lower in the active RA (23.67%, range 8.15–46.72) as compared to the clinical remission group (57.24%, range 27.72–90.72) and healthy controls (64.18%, range 59.66–66.97), *p* < 0.0001 (95% CI 20.88–44.01) and *p* < 0.0001 (95% CI 33.54–47.30), respectively (Fig. 2B). When Foxp3 Treg inhibitory activity was compared within the same patient, inhibitory activity in active RA state (12.2 ± 4.6) was lower than the remission state (39.9 ± 11.9), *p* value < 0.05 (Supplementary Figure S3B). Moreover, analysis of percent inhibition according to the DAS28 score showed the Treg inhibitory activity is lowest in high disease activity patients (Fig. 2C). For cross-tabulation analysis, a cutoff of Treg inhibitory activity for separating patient-outcome was 35%, which was calculated from the mean difference of percent inhibition between active RA and remission state (Fig. 2D). Sensitivity, specificity, PPV, NPV, LR+ and LR− were 85.71% (95% CI 57.19–98.22%), 92.31% (95% CI 63.97–99.81%), 92.31% (95% CI 64.33–98.76%), and 85.71% (95% CI 62.22–95.62%), 11.14 (1.67–74.14), and 0.15 (0.04 to 0.56), respectively. In addition, an inverse relationship of Treg inhibitory activity and DAS28 and VAS were demonstrated (Fig. 2E, F). Furthermore, the Treg inhibitory activity was observed in RA patients who were classified based on their received doses of MTX. Percent Treg inhibitory activity (Median ± IQR) in patient who received < 10 mg/week (low dose), 11–17.5 mg/week (intermediate dose) and ≥ 20 mg/week (high dose) are 49.4 ± 20.7, 35.2 ± 25.4 and 18.4 ± 10.6, respectively. We found that Treg activity in < 10 mg/week MTX group is higher than in the other groups. A significant difference was found when compared to ≥ 20 mg/week MTX group (*p* < 0.01) (Supplementary Figure S4A). Besides, the RA patients who received high and intermediate doses of MTX had more DAS28 score when compared to the low dose group (*p* < 0.05) (Supplementary Figure S4B). Taken together, the lowest Treg inhibitory activity could be found in the RA patients who had high disease-severity.

### Plasma cytokines

Among the 14 plasma cytokines (IL-2, IL-4, IL-5, IL-6, IL-9, IL-10, IL-13, IL-17A, IL-17F, IL-21, IL-22, INF-γ, TGF-β, and TNF-α) analyzed by using flow-cytometry, the levels of IL-6, IL-10, IL-17A, and TNF-α were significantly lower in clinical remission as compared to the active RA group. The levels of those four cytokines and TGF-β in active patient group were significantly higher than healthy control group. Also, the TGF-β in the remission group was significantly higher than those of healthy controls (Table 2). Box Plot graphic of the significant cytokines is given in Supplementary Figure S5. When compared to the DAS28 scores, the IL-10 and TGF-β level were significantly lower between the remission group and low (≥ 2.6–3.2) DAS28 score group (46.2 ± 14.1 vs. 63.4 ± 79.3 pg/mL (*p* value < 0.05) and 127.1 ± 104.4 vs. 402.4 ± 262.3 ng/mL (*p* value < 0.01) respectively), and IL-10 and IL-17A levels were significantly lower between the remission and the moderate (≥ 3.2–5.0) DAS28 score groups (46.2 ± 14.1 vs. 55.9 ± 24.8 pg/mL (*p* value < 0.05) and 1.9 ± 2.5 vs. 6.7 ± 9.5 pg/mL (*p* value < 0.05), respectively (Supplementary Figure S1). Besides, the other cytokines (IL-2, IL-4, IL-5, IL-9, IL-13, IL-17F, IL-21, IL-22 and INF-γ) are not significantly difference among these groups comparison.

## Discussion

Previously, a study in Lupus patients demonstrated that Treg associated with immunological remission of Lupus^14^. However, no study has been discovered in RA patients, in terms of indicating immunological remission status. In our previous study, the method of Treg inhibitory detection was validated for studying the peripheral blood Treg inhibitory function in RA^12^. However, no study has used the Treg inhibitory function to demonstrate the immunological tolerance in remission RA.

Foxp3+ Treg properties may reflect the prognosis or prediction of alteration of clinical symptom of RA patients. This study shows that the number of peripheral blood Foxp3+ Treg cells is significantly lower in clinically active as compared to remission RA patients while both demonstrate lower quantity than that of the healthy controls, which is in accordance with the other studies^5^. A previous study reported a negative correlation of Foxp3+ Treg number and DAS28 score in RA patients^15^. However, in our study, when the patients were separated based on their DAS28 score, the number of Foxp3+ Treg cells is significantly decreased only in the moderate DAS28 group (≥ 3.2–5.0) compared to remission RA (Supplementary Figure S1). We discuss that not only the number of DAS28 high group (n = 2) is limitation of this study, but also the plasticity of Treg might also be a critical factor of the Foxp3+ measurement^16^. Nevertheless, the Treg function of high DAS28 group is clearly decreased when compared to low and moderate groups (Fig. 2C). Moreover, the negative correlation between the Treg inhibition *vs* VAS (*r* = −0.60) or DAS28 score (*r* = −0.513) was found in this study (Fig. 1E, F). These correlations clarified that Treg function might correlated with clinical severity rather than the number of Foxp3+ Treg.

Accordingly, The sole numbers of Foxp3+ Treg cells may be insufficient to determine the status of immune suppression in RA patients because Treg plasticity can occur at sites of inflammation^6^. Myeloid-derived suppressor cells (CD14+ HLA-DR-low MDSCs) and cytokines, such as IL-1β, IL-6, IL-17, and TGF-β, are involved in the inter-conversion between Foxp3+-and Th17-Treg cells^6,17^. In addition, increased numbers of IL-17-producing Treg cells are present in RA patients’ peripheral blood as compared to healthy individuals^18^. Although this study does not measure Th17-Treg cell numbers, the decreased Foxp3+ Treg: CD4+CD25+CD127+ cell ratio (termed Foxp3+ Treg ratio) was observed in most RA patients, especially in active RA when compared to healthy individuals (Table 2). The CD4+CD25+CD127+ population indicates not only activating T cells but also effector memory T cells^19^. Such finding additionally indicated an increase in numbers of other T or effector T cells and/or a decrease in Foxp3+ Treg numbers occurring in RA patients. In addition, a significant correlation of Foxp3+ Treg ratio with CD4+CD25high+CD127low−:CD4+CD25+CD127+ cell ratio (*r* = 0.878, *p* value < 0.0001; Supplementary Figure S2) was found in this study. The correlation indicated that using either the intracellular (Foxp3) or extracellular markers for Treg identification can demonstrate the imbalance of Treg and/or effector T cells in RA patients. Moreover, the imbalance of Treg and others T cell was reported in other autoimmune diseases^20^.

Co-culture inhibition assay of Treg cells has been used for determining the function of Treg in many diseases^7,21,22^. Foxp3+ Treg inhibitory activity in such system was lowest in active RA patients and lower in the remission RA patients than the healthy individuals. The inhibitory activity in patients with DAS28 score ≤ 2.6 is significantly higher than patients with moderate and high DAS28 scores (> 3.2). Furthermore, the lowest Treg inhibitory activity was observed in patients who received high dose of MTX (≥ 20 mg/week) when compared to low dose and moderate dose MTX used patients. This finding also related to the severity of disease. Because the patients who received high dose of MTX had high DAS28 while the patients with low DAS28 score had low dose MTX used (Supplementary Figure S4B). However, these patients had different combined-DMARDs used. Currently, no study have tested the effect of MTX on functional peripheral blood Treg isolated from of RA patient. However, an in vitro study shown that MTX has no effects on Foxp3+ expression in healthy controls. Perhaps, the study in vitro may not demonstrating the effect of MTX in RA patient because drug mechanism is different when compared to in vivo study^23^. Also, the Foxp3 expression study is not enough for indicating the Treg function because of T cell plasticity^16^.

In terms of indicating the immunological remission, this study shows high sensitivity (85.71%), specificity (92.31%), LR+ (11.14) and LR− (0.15) of Treg inhibitory activity to differentiate between the remission and active status. As an illustration, those patients with ≥ 35% Treg inhibitory activity are in remission state. In contrast, those with Treg activity less than 35%, are in active state RA.

Previous studies showed slightly impaired inhibitory activity in both early active and active RA patients, which is not significantly different from those well-controlled RA patients^7,8^. These observations might be due to a small number of patients studied and that Treg isolation is performed using only CD4 and CD25 markers. Ehrenstein et al.^24^ noted that suppressive function is recovered in RA patients following anti-TNF therapy, inhibiting IFN-γ and TNF-α secretion from autologous effector T cells. In this study, Foxp3+ Treg inhibition property is determined in the same RA patients in both active and remission states following DMARDs treatment. The results clearly show a decreased inhibitory activity of Foxp3+ Treg when RA clinical status alters from remission to active (relapsed) disease. In a follow-up study, it is reported that the Treg number is improved in patients treated with conventional and biological DMARDs. However, the Treg inhibitory activity is not mentioned^15,25^.

Treg function in RA also depends on the presence of a variety of pro-inflammatory cytokines^26^. That is the reason why this study also measured the plasma cytokines. According to our results, the levels of IL-6, IL-10, IL-17A, and TNF-α are elevated in patients with active RA, with significantly higher IL-10 and IL-17A level in patients in the low and moderate disease activity, respectively, as compared to those in remission. While inhibitory function of Treg is decreased in active RA. Reasonably, A study shown that TNF-α binds to Treg tumor necrosis factor receptor type II and down-regulates FOXP3 expression by inducing the expression of protein phosphatase 1. This specifically dephosphorylates Ser418, leading to impaired FOXP3 phosphorylation, and thereby loss of Treg function^26^. IL-10 is an anti-inflammatory cytokine that is secreted by Treg and released as a consequence of high levels of inflammatory cytokines. As an evidence, a supporting study shows that amounts of mRNA expression of IL-10 cytokine family from the PB and synovial fluid mononuclear cells in RA were increased^27^. This is not a surprising finding that the higher levels of IL-6 and IL-17A are found in the active disease state. Both of them are essential mediators for auto-antibody production and Th17 differentiation^28^. Differing from the healthy controls, no significant difference is discerned about TGF-β level between the active and remission RA patients although a slightly but significantly higher level is observed between low disease activity and remission. This finding is well supported by a result from a previous study illustrating that TGF-β signaling in RA synovial tissue is increased in a murine collagen-induced arthritis model^29^. Although, only DAS28 high group (N = 2) is limitation of this study for measured the plasma cytokines, but the level of every cytokines are same approach in between active RA and remission RA groups.

In terms of serology for indicating the immunological remission, this study demonstrated that ACPA/RF positivity do not correlate with DAS28 score (r = 0.311, *p* value = 0.115). However, all patients with DAS28 ≥ 3.2–5.0 are having ACPA/RF positivity (Supplementary Table 1). A two-year follow-up study on RA has suggested that levels of ACPA and RF do not associate with RA outcome. At one year follow-up, ACPA and RF levels decrease only by 31 and 56%, respectively^3^. In a large cohort survey, ACPA and RF levels are only moderately correlated with the markers of inflammation^30^. A randomized study demonstrates that the ACPA and RF levels are not predictive of joint damage progression in early RA patients^31^. Recently, a multi-biomarker disease activity (MBDA) scoring system has been developed. This score can be used to monitor the disease activity over time. Despite its significant correlation with DAS28, MBDS scores requires measurement of 12 serum proteins as contributing parameters for score calculation^32^.

In summary, this study demonstrates that the degree of Foxp3+ Treg inhibitory activity of RA patients in remission is higher than that of active RA. As well, it is inversely correlated to the disease activity score-28 in patients with moderate and high disease severity. Moreover, it also provides a high specificity and LR+ for indicating the remission status in RA patients. TGF-β and IL-10 levels are significantly elevated in active state. In addition, the number of Foxp3+ Treg cells and Foxp3+ Treg ratio are higher in remission as compared to active state. Taken altogether, Foxp3+ Treg inhibitory activity reflects the restoration of the immune system imbalance in patients with active RA, rendering its potential as a prognostic marker and representative of immunological remission in RA. There may be some possible limitations in this study, the age median of healthy control is younger than the remission RA and active RA patients which probably influences the human peripheral blood naturally occurring Treg declines with age including the cytokine expression pattern called inflamm-aging that underpin most major age-related disease. Fairly, for indicating the immunological remission status, we mainly focus on the Treg inhibitory activity of the remission RA and active RA patients in which the age-range of these groups is the same. However, further study involving a larger cross-sectional or cohort as well as age-matched control are warranted given the promising results achieved.

## Supplementary information

Supplementary information.

## References

[1] Fransen J, Stucki G, van Riel PLCM (2003). Rheumatoid arthritis measures: Disease Activity Score (DAS), Disease Activity Score-28 (DAS28), Rapid Assessment of Disease Activity in Rheumatology (RADAR), and Rheumatoid Arthritis Disease Activity Index (RADAI). Arthritis Care Res..

[2] Schett G (2016). Tapering biologic and conventional DMARD therapy in rheumatoid arthritis: Current evidence and future directions. Ann. Rheum. Dis..

[3] Ursum J, Bos WH, van Dillen N, Dijkmans BA, van Schaardenburg D (2010). Levels of anti-citrullinated protein antibodies and IgM rheumatoid factor are not associated with outcome in early arthritis patients: A cohort study. Arthritis Res. Ther..

[4] Morita T (2016). The proportion of regulatory T cells in patients with rheumatoid arthritis: A meta-analysis. PLoS ONE.

[5] Oh S, Rankin AL, Caton AJ (2009). CD4+CD25+ regulatory T cells in autoimmune arthritis. Immunol. Rev..

[6] Kleinewietfeld M, Hafler DA (2013). The plasticity of human Treg and Th17 cells and its role in autoimmunity. Semin. Immunol..

[7] Lawson CA (2006). Early rheumatoid arthritis is associated with a deficit in the CD4+CD25 high regulatory T cell population in peripheral blood. Rheumatology.

[8] van Amelsfort JMR, Jacobs KMG, Bijlsma JWJ, Lafeber FPJG, Taams LS (2004). CD4+CD25+ regulatory T cells in rheumatoid arthritis: Differences in the presence, phenotype, and function between peripheral blood and synovial fluid. Arthritis Rheumatism.

[9] Samson M (2012). Brief report: Inhibition of interleukin-6 function corrects Th17/Treg cell imbalance in patients with rheumatoid arthritis. Arthritis Rheum..

[10] Kmieciak M (2009). Human T cells express CD25 and Foxp3 upon activation and exhibit effector/memory phenotypes without any regulatory/suppressor function. J. Transl. Med..

[11] Liu W (2006). CD127 expression inversely correlates with FoxP3 and suppressive function of human CD4+ T reg cells. J. Exp. Med..

[12] Kanjana K, Paisooksantivatana K, Matangkasombut P, Chevaisrakul P, Lumjiaktase P (2019). Efficient short-term expansion of human peripheral blood regulatory T cells for co-culture suppression assay. J. Immunoassay Immunochem..

[13] Kay J, Upchurch KS (2012). ACR/EULAR 2010 rheumatoid arthritis classification criteria. Rheumatology.

[14] Zhang L, Bertucci AM, Ramsey-Goldman R, Burt RK, Datta SK (2009). Regulatory T cell (Treg) subsets return in patients with refractory lupus following stem cell transplantation, and TGF-β-producing CD8^+^ Treg cells are associated with immunological remission of lupus. J. Immunol..

[15] Abaza N, El-kabarity RH, Abo-Shady RA (2013). Deficient or abundant but unable to fight? Estimation of circulating FoxP3+ T regulatory cells and their counteracting FoxP3− in rheumatoid arthritis and correlation with disease activity. Egypt. Rheumatol..

[16] Jung MK, Kwak J-E, Shin E-C (2017). IL-17A-producing Foxp3(+) regulatory T cells and human diseases. Immune Netw..

[17] Hoechst B, Gamrekelashvili J, Manns MP, Greten TF, Korangy F (2011). Plasticity of human Th17 cells and iTregs is orchestrated by different subsets of myeloid cells. Blood.

[18] Wang T (2015). Regulatory T cells in rheumatoid arthritis showed increased plasticity toward Th17 but retained suppressive function in peripheral blood. Ann. Rheum. Dis..

[19] Huster KM (2004). Selective expression of IL-7 receptor on memory T cells identifies early CD40L-dependent generation of distinct CD8+ memory T cell subsets. Proc. Natl. Acad. Sci. USA.

[20] Lee GR (2018). The balance of Th17 versus Treg cells in autoimmunity. Int. J. Mol. Sci..

[21] Wehrens EJ (2011). Functional human regulatory T cells fail to control autoimmune inflammation due to PKB/c-akt hyperactivation in effector cells. Blood.

[22] Santagata S (2017). Targeting CXCR4 reverts the suppressive activity of T-regulatory cells in renal cancer. Oncotarget.

[23] Oh JS (2013). The effect of various disease-modifying anti-rheumatic drugs on the suppressive function of CD4+CD25+ regulatory T cells. Rheumatol. Int..

[24] Ehrenstein MR (2004). Compromised function of regulatory T cells in rheumatoid arthritis and reversal by anti-TNFα therapy. J. Exp. Med..

[25] Szalay B (2014). The impact of conventional DMARD and biological therapies on CD4+ cell subsets in rheumatoid arthritis: A follow-up study. Clin. Rheumatol..

[26] Nie H (2013). Phosphorylation of FOXP3 controls regulatory T cell function and is inhibited by TNF-α in rheumatoid arthritis. Nat. Med..

[27] Alanärä T, Karstila K, Moilanen T, Silvennoinen O, Isomäki P (2010). Expression of IL-10 family cytokines in rheumatoid arthritis: Elevated levels of IL-19 in the joints. Scand. J. Rheumatol..

[28] Alunno A, Carubbi F, Giacomelli R, Gerli R (2017). Cytokines in the pathogenesis of rheumatoid arthritis: New players and therapeutic targets. BMC Rheumatol..

[29] Gonzalo-Gil E (2013). Transforming growth factor (TGF)-β signalling is increased in rheumatoid synovium but TGF-β blockade does not modify experimental arthritis. Clin. Exp. Immunol..

[30] Ursum J, Bos WH, van de Stadt RJ, Dijkmans BAC, van Schaardenburg D (2009). Different properties of ACPA and IgM-RF derived from a large dataset: further evidence of two distinct autoantibody systems. Arthritis Res. Ther..

[31] Hafström I (2014). Rheumatoid factor and anti-CCP do not predict progressive joint damage in patients with early rheumatoid arthritis treated with prednisolone: A randomised study. BMJ Open.

[32] Hirata S (2013). A multi-biomarker score measures rheumatoid arthritis disease activity in the BeSt study. Rheumatology.

