# Supplementary material for: Inhibitory activity of FOXP3+ regulatory T cells reveals high specificity for displaying immune tolerance in remission state rheumatoid arthritis
Source: Sci Rep. 2020 Nov 13;10:19789. doi: 10.1038/s41598-020-76168-1 (PMC7666192; doi:10.1038/s41598-020-76168-1)
Supplement: Supplementary file 1 — Supplementary information. [file 41598_2020_76168_MOESM1_ESM.docx]

**Inhibitory Activity of FOXP3+ Regulatory T Cells Reveals High Specificity for Displaying Immune Tolerance in Remission State Rheumatoid Arthritis.**

Korawit Kanjana^1, 4^, Parawee Chevaisrakul^2^, Ponpan Matangkasombut^3^,

Karan Paisooksantivatana^1^, Putthapoom Lumjiaktase^1^

**Affiliations**

^1^Department of Pathology, Faculty of Medicine, Ramathibodi Hospital, Mahidol University, Bangkok, Thailand

^2^Division of Allergy, Immunology and Rheumatology, Department of Medicine, Faculty of Medicine, Ramathibodi Hospital, Mahidol University Bangkok, Thailand

^3^Department of Microbiology, Faculty of Science, Mahidol University, Bangkok, Thailand

^4^Center for Immunology and Inflammatory Diseases, Division of Rheumatology, Allergy and Immunology, Massachusetts General Hospital, Harvard Medical School, Boston, MA, United States

**Supplementary data**

**Supplementary Table 1**. DAS28 score between rheumatoid arthritis (RA) patients with negative and positive serology.

|  | DAS score in patients with different serology status | | *p-*value |
| --- | --- | --- | --- |
|  | Negative (%) | Positive (%) |  |
| DAS28 score (all patients, n = 27) | 2.3 ± 0.7 (26) | 3.2 ± 1.4 (74) | 0.111 |
| DAS28 score in remission RA group (n = 14) | 2.1 ± 0.7 (36) | 2.1 ± 0.6 (64) | 0.955 |
| Remission DAS28 score (<2.6) (n = 11) | 2.0 ± 0.7 (36) | 1.9 ± 0.6 (64) | 0.899 |
| Low DAS28 score (≥2.6-3.2) (n = 8) | 2.6 ± 0.0 (16) | 2.8 ± 0.2 (83) | 0.541 |
| Moderate DAS28 score (≥3.2-5.0) (n = 6) |  | 4.0 ± 0.5 (100) | - |
| High DAS28 score (≥5.0) (n = 2) |  | 5.8 ± 0.9 (100) | - |

*Significance at *p*-value <0.05, DAS28; score disease activity score-28, Serology test defined by ACPA (anti-citrullinated protein antibodies) and/or RF (rheumatoid factor) positive.


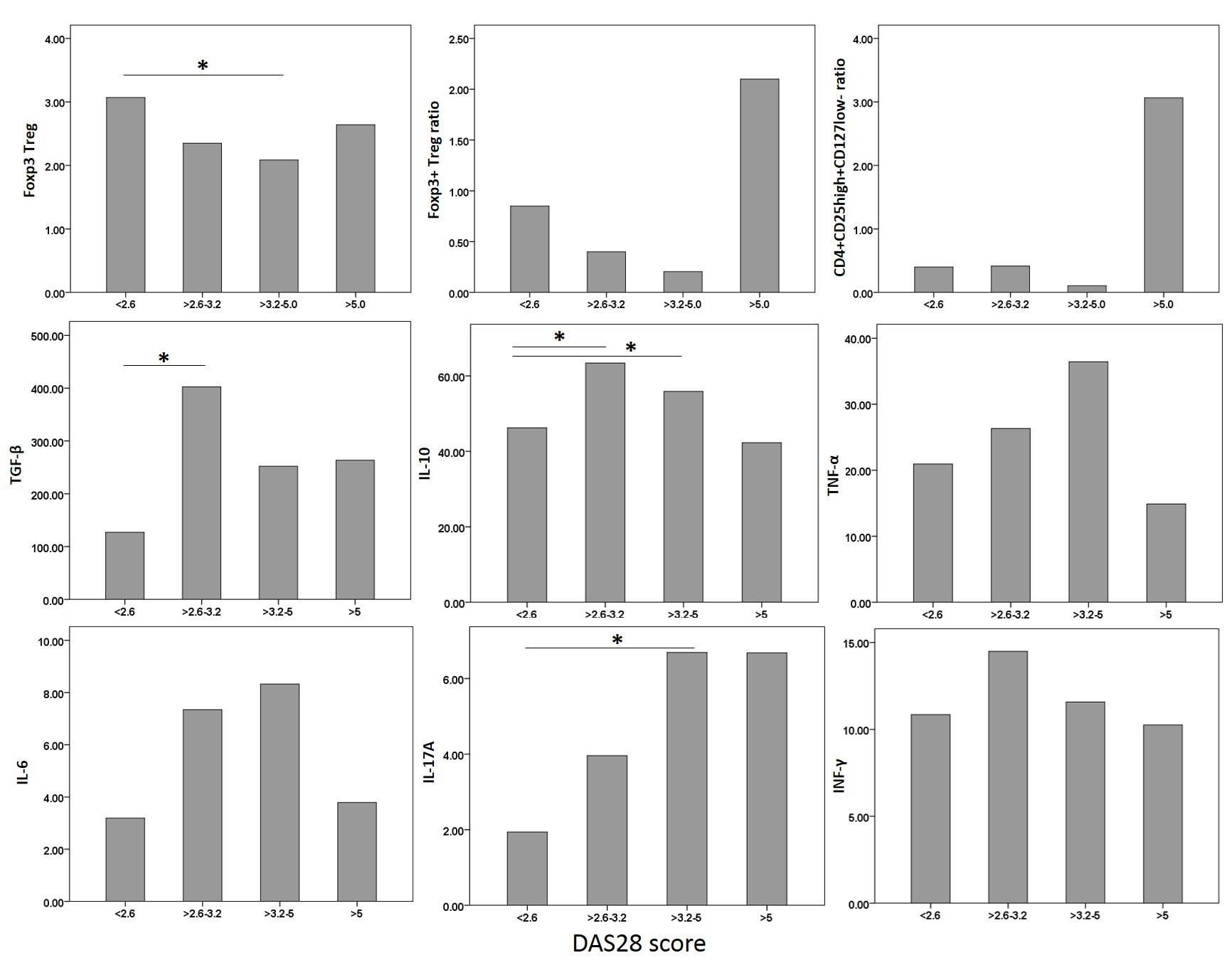


**Supplementary Figure S1** The immunological biomarkers in a variety of DAS28 scores

Classification: <2.6; remission (n=11)**,** >2.6-3.2; low disease activity (n=8), >3.2-5.0; moderate DAS (n=6)**,** >5.1; high DAS (n=2), TGF-β; Transforming growth factor beta, IL; Interleukin, INF-γ; Interferon gamma, **p*-value <0.05 (Mann–Whitney *U* test)

**
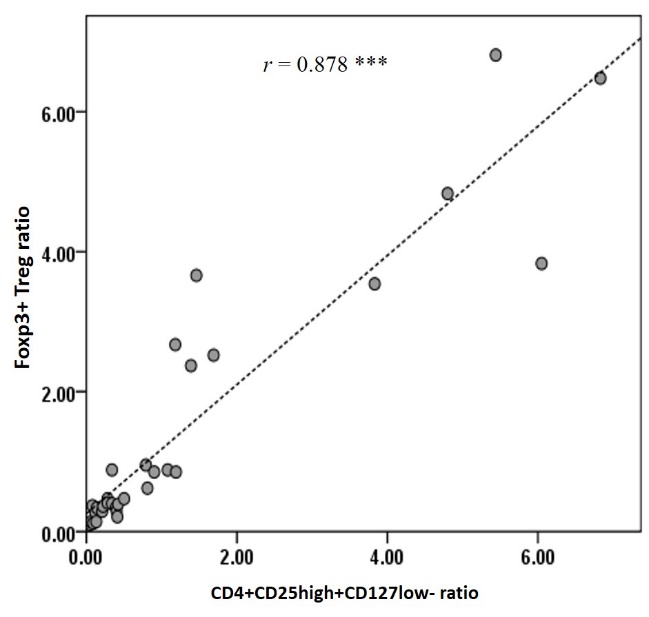
**

**Supplementary Figure S2** The correlation between Foxp3+ Treg ratio and CD4+CD25high+CD127low- ratio. Both ratios are having positive correlation (r = 0.878), *** *p*-value <0.0001 (Person correlation test)


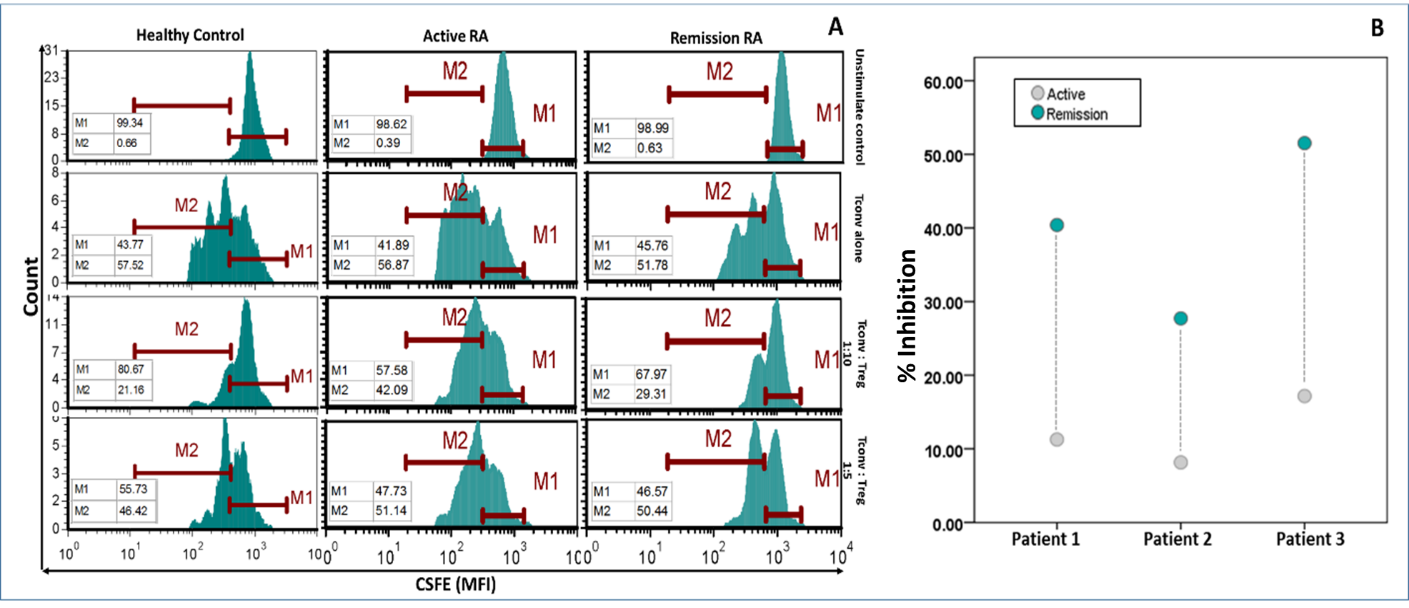


**Supplementary Figure S3.** (A) Histograms analysis and mean fluorescent index (MFI) of CFSE for measuring the proliferation of CFSE-Tconv by flow-cytometry. This figure demonstrates an example the MFI of CFSE-Tconv from co-culture inhibition assay of each state of RA and normal individual in co-cultivation of Tconv:Treg ratio at 1:10 and 1:5. Unstimulate control; Tconv alone without stimulation, Tconv alone; Tconv was stimulated by anti-human CD3/CD28 beads, Tconv: Treg is a ratio of cell co-cultivation. M1; marker 1 from undivided cell or original division, M2; proliferated Tconv. The % inhibition of Treg was calculated by the formula; please see in method. (B) The alteration of percentage inhibition of the individual same person (*n*=3). The result show the inhibitory activity of patients with active state RA was increased when they enter to the remission; 12.19 ± 4.58 to 39.88 ± 11.91, respectively). Patient no.1 was treated by MTX 7.5 mg/wk LEF 40 mg/wk, patient no.2 was treated by MTX 2.5 mg/wk SSZ 250 mg/day AZA 50 mg/wk, and the patient no.3 was treated by SSZ 1000 mg/day LEF 20 mg/wk MTX 10 mg/wk. Abbreviation, MTX; methotrexate, LEF; leflunomide, SSZ; sulfasalazine, AZA; azathioprine.


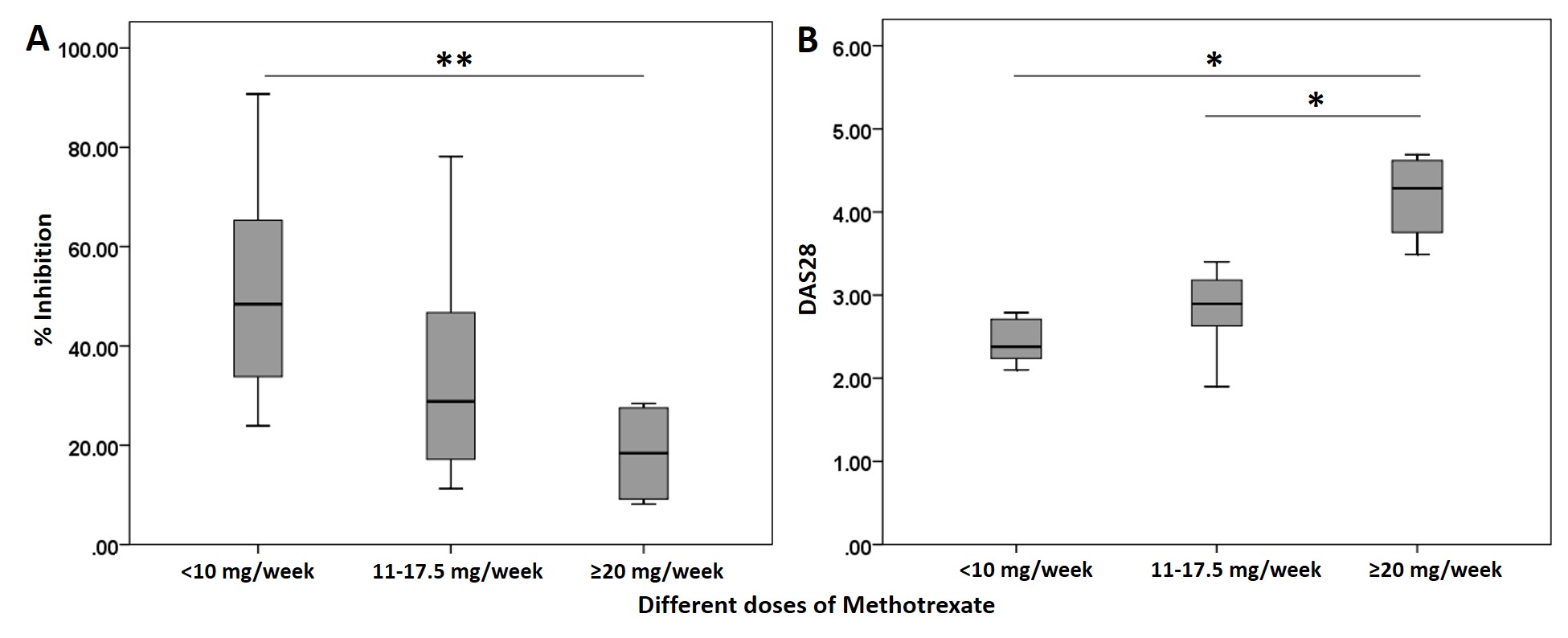


**Supplementary Figure S4**. Percent Treg inhibition (A) and DAS28 score (B) in RA patients which categorized based on different doses of Methotrexate used including; <10 mg/week (n=17), 11-17.5 mg/week (n=6) and ≥20 mg/week (n=4). **p*-value <0.05, ** *p*-value <0.01. (Mann–Whitney *U* test)


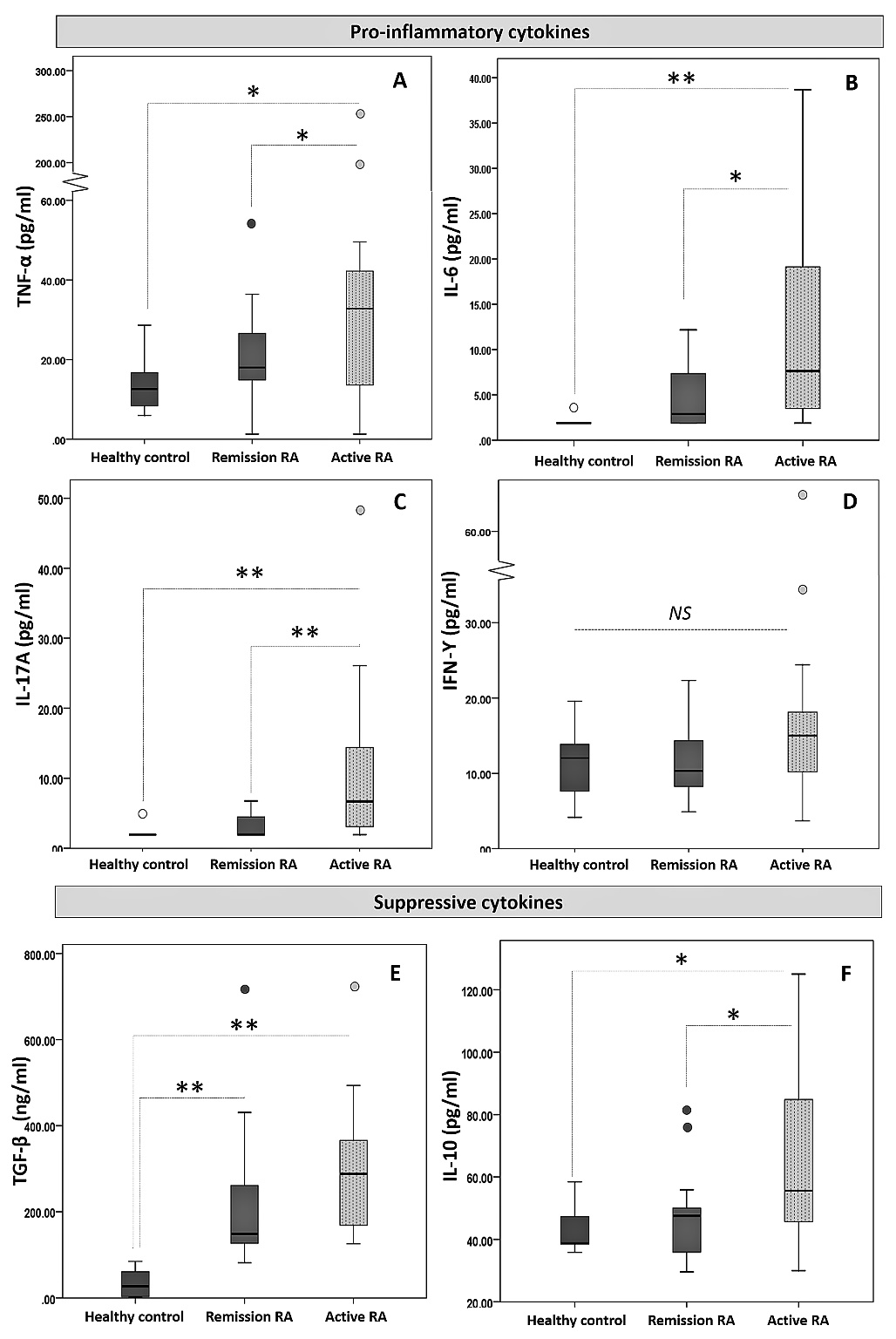


**Supplementary Figure S5**. Plasma cytokines of different states of RA patients (*n*=14; remission, *n*=13; active state) and healthy controls (*n*=5) were determined by flow-cytometry. (A) TNF-α, (B) IL-6, (C) IL-17A and (D) INF-γ were categorized as pro-inflammatory cytokines whereas TGF-β (E) and IL-10 (F) were categorized as suppressive cytokines in both states of RA and healthy control. **p*-value <0.05, ** *p*-value <0.01, *** *p*-value <0.0001, and NS; no significant difference. (Mann–Whitney *U* test)
